# Esophageal Clearance in Laryngopharyngeal Reflux Disease: Correlation of Reflux Scintigraphy and 24-hour Impedance/pH in a Cohort of Refractory Symptomatic Patients

**DOI:** 10.4274/mirt.galenos.2019.30085

**Published:** 2020-02-17

**Authors:** Leticia Burton, Gregory L. Falk, Karl Baumgart, John Beattie, Scott Simpson, Hans Van der Wall

**Affiliations:** 1 University of Notre Dame, CNI Molecular Imaging, Sydney, Australia; 2 Concord Hospital and University of Sydney, Sydney Heartburn Clinic, Sydney, Australia; 3 North Shore Medical Centre, Sydney, Australia; 4 Ryde Medical Centre, Sydney, Australia; 5 Sydney Adventist Hospital and University of Sydney, Sydney, Australia

**Keywords:** Gastroesophageal reflux disease, laryngopharyngeal reflux, reflux, impedance, pH, manometry, scintigraphy, pulmonary aspiration

## Abstract

**Objectives:**

The role of gastroesophageal reflux disease (GERD) in the aetiology of laryngopharyngeal reflux (LPR) is poorly understood and remains a controversial issue. The 24-hour impedance monitoring has shown promise in the evaluation of LPR but is problematic in pharyngeal recording. We have shown the utility of scintigraphic studies in the detection of LPR and lung aspiration of refluxate. Correlative studies were obtained in patients with a strong history of LPR and severe GERD.

**Methods:**

A highly selected sequential cohort of patients with a high pre-test probability of LPR/severe GERD who had failed maximal medical therapy were evaluated with 24-hour impedance/pH, manometry and scintigraphic reflux studies.

**Results:**

The study group comprised 34 patients (15 M, 19 F) with a mean age of 56 years (range: 28-80 years). The majority had LPR symptoms (mainly cough) in 31 and severe GERD in 3. Impedance bolus clearance and pH studies were abnormal in all patients in the upright and supine position. A high rate of non-acid GERD was detected by impedance monitoring. LOS tone and ineffective oesophageal clearance were found in the majority of patients. Scintigraphic studies showed strong correlations with impedance, pH and manometric abnormalities, with 10 patients showing pulmonary aspiration.

**Conclusion:**

Scintigraphic studies appear to be a good screening test for LPR and pulmonary aspiration as there is direct visualisation of tracer at these sites. Impedance studies highlight the importance of non-acidic reflux and bolus clearance in the causation of cough and may allow the development of a risk profile for pulmonary aspiration of refluxate.

## Abstract (tr)

**Amaç:**

Laringofarengeal reflü (LFR) etiyolojisinde gastroözofageal reflü hastalığının (GÖRH) rolü tam olarak anlaşılamamıştır ve bu durum tartışmalı bir konu olmaya devam etmektedir. Yirmi dört saatlik empedans monitörizasyonu LFR’nin değerlendirilmesinde umut vaat etmekle birlikte faringeal kayıtlamada sorun yaşanmaktadır. Bu çalışmada LFR’nin ve akciğer aspirasyonunun saptanmasında sintigrafik çalışmaların yararı gösterilmiştir ve şiddetli LFR tarif eden ve ileri derecede GÖRH öyküsü olan hastalarda korelasyon çalışmaları yapılmıştır.

**Yöntem:**

Maksimum medikal tedaviye yanıt vermeyen ve ön testte LFR/şiddetli GÖRH olasılığı yüksek saptanan seçilmiş bir hasta grubu; 24 saatlik empedans/pH monitörizasyonu, manometri ve sintigrafik reflü çalışmaları ile değerlendirildi.

**Bulgular:**

Çalışma grubu, 15’i erkek, 19’u kadın olmak üzere 34 hastadan oluşmaktaydı ve yaş ortalaması 56 (28-80) idi. Hastaların 31’inde LFR semptomları (çoğunlukla öksürük) ve 3’ünde şiddetli GÖRH semptomları vardı. Empedans bolus klirensi ve pH çalışmaları, ayakta ve sırtüstü pozisyonda tüm hastalarda anormaldi. Empedans monitörizasyon ile yüksek oranda asidik olmayan GÖRH saptandı. Hastaların çoğunda düşük özofageal sfinkter tonusu ve inefektif özofageal klirens saptandı. Sintigrafik çalışmalar pulmoner aspirasyon gelişen 10 hastada; empedans, pH ve manometrideki anormalliklerle yüksek korelasyon gösterdi.

**Sonuç:**

Sintigrafik çalışmalar, LFR ve pulmoner aspirasyon için iyi bir tarama testi gibi görünmektedir, çünkü bu bölgelerde tracerin doğrudan gösterilmesi mümkündür. Empedans çalışmaları, öksürüğün nedeni olarak asidik olmayan reflü ve bolus klirensinin önemini vurgulamaktadır ve reflünün pulmoner aspirasyonu için bir risk profilinin geliştirilmesinde kullanılabilir.

## Introduction

The pathophysiology of proximal gastrooesophageal reflux disease (GERD) causing laryngopharyngeal reflux (LPR) is poorly understood (1,2). It is an important consideration in the aetiology of chronic cough which remains undiagnosed after eight weeks of specialist investigation (1,2,3,4). The pathophysiology of reflux-induced cough is poorly described and the disease remains in dispute (1,2). Various disease processes may be generators of laryngeal and pharyngeal symptoms including proximal GERD. These may manifest as pharyngeal reflux, laryngeal contamination and pulmonary aspiration as well as acid reflex-mediated bronchospasm (5,6).

Response to proton pump inhibitor (PPI) therapy has been utilised as a diagnostic test (7,8) as there has been no accurate diagnostic test for LPR by which to make the initial diagnosis and to interrogate the success of treatment. A high placebo response in treatment of cough makes the matter more complex when evaluating therapy (7). Investigation of this situation by 24-hour pH reflux testing has been bedevilled by artefacts in the pharynx (9), leading to attempts to modify instrumentation to increase accuracy and reproducibility. The newer technology of reflux impedance monitoring has shown potential to identify non-acidic and slightly acidic reflux episodes as well as pharyngeal contamination (9,10). Intra-observer variability however has been a problem for accuracy of pharyngeal readings (10,11). Identifying reflux high in the oesophagus where observations are more accurate than in the pharynx does not necessarily predict pharyngeal exposure, as the upper oesophageal sphincter separates the chambers. The issue of an episode of reflux changing acidity during ascent in the oesophagus confounds proximal pH measurements, as does the recognition of symptoms associated with non-acid reflux (10,12).

Reflux scintigraphy has been utilised in children and to a variable extent in adults to evaluate pharyngeal contamination and pulmonary aspiration of refluxate (13,14,15). There have however been multiple technical difficulties and a lack of standardisation between studies with variable and sometimes contradictory results (13,16,17,18). We have developed and validated a consistent scintigraphic technique for the detection of GERD and LPR with good correlations with pH monitoring and manometry (19,20).

We hypothesised that scintigraphic reflux studies could provide additional information and complement 24-hour pH and impedance studies in patients with GERD and suspected LPR. A secondary purpose of the study was to evaluate impedance reflux studies in prediction of proximal reflux disease causing LPR symptoms and lung aspiration of refluxate.

## Materials and Methods

### Clinical

Consecutive patients failing adequate medical investigation and management, with a high pre-test probability of proximal GERD with LPR symptoms were referred to a tertiary anti-reflux surgical service in the past 3 years. Patients underwent standard symptom pro-forma interview with regard to LPR symptoms including amongst others, cough, sore throat, voice change, and dyspnoea. Patients had previously undergone gastroscopy and laryngoscopy for symptoms of GERD/LPR. Alternative causes of LPR symptoms had been excluded by multi-disciplinary investigations.

Manometry, 24-hour dual channel pH and impedance reflux and scintigraphic reflux studies were obtained in all patients while off PPI therapy.

Hiatus hernia was diagnosed by endoscopy.

Oesophageal manometry was performed under topical nasal anaesthesia using a dent sleeve 4 mm trans-nasal 6 lumen catheter placed by identification of the lower oesophageal sphincter (LOS) by pull through and placement of the sleeve in the LOS. Wet swallows (10) of 2.5 mL water were performed by stationary technique using the dent mark 2 infusion pump (Dentsleeve International Ltd., Mississauga, Ontario, Canada). Studies were performed in the supine position. The swallows were assessed for peristaltic efficacy (21) and sphincter characteristics were determined. A lesser sub-group of motility disturbance was created for 20-30% ineffective oesophageal motility (IEM) which would previously have been included in the normal group. Resting pressure of the sphincter and nadir pressure were reported from the mid-end expiratory pressure.

Twenty four-hour impedance reflux study with two channel 24-hour pH was performed after cessation of all anti-acid therapy for 48 hours. Patients were prepared with local anaesthetic prior to insertion of a trans-nasal catheter consisting of 2 level impedance rings and 2 level pH electrodes connected to an external monitoring device. Standard calibration was carried out. Impedance rings were placed at 5 and 15 cm above the upper border of the LOS (Zephyr device, catheter ZAI-BD31, Sandhill Co, Highlands Ranch, Colorado, USA). There were no dietary restrictions during the testing period other than ingestions of acidic beverages. Catheter placement was ascertained by measurements taken at manometry with the lower pH electrode 5 cm above the upper border of the LOS, the upper, 15 mm higher. The patient returned the following day when the assembly was removed. Meal-times were included in the reporting analysis. Reports of 24-hour pH and 24-hour impedance reflux were then generated using autoscan and manual review. Events which were considered not to be reflux or indeterminate were excised from the report. The categories of reflux were classified according to the consensus on impedance and pH monitoring (22). Briefly, it was based on oesophageal pH during reflux detected by impedance monitoring. Acid reflux was a fall in pH below 4, weakly acid reflux was a fall in pH which was ≥4 but ˂7 and non-acid reflux where oesophageal pH increases ≥7 or remained ≥7 during reflux. Liquid bolus entry was the time when the 50% fall in impedance from baseline during liquid reflux was reached. Bolus duration was the time from liquid bolus entry to liquid bolus clearance (impedance increasing for >5 seconds).

### Scintigraphy

**Figure 1:**
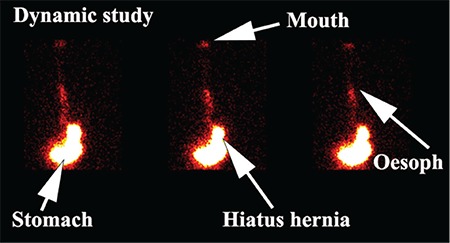
Dynamic scintigraphic study. The sequence of images demonstrates tracer activity in the stomach with evidence of a hiatus hernia and gastro-oesophageal reflux to the level of the oropharynx. Note the progressive accumulation of tracer in the region of the oropharynx (mouth)

**Figure 2:**
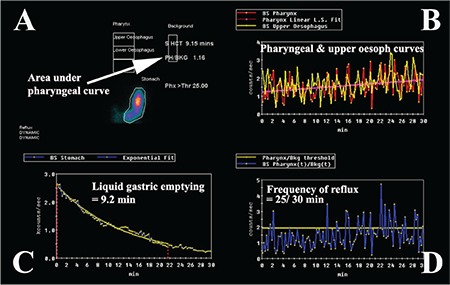
Graphical analysis of the dynamic study. (A) This panel demonstrates the regions of interest over the pharynx, upper and lower oesophagus with the background region of interest. It also indicates the area under the pharyngeal curve. Panel (B) illustrates the graphical output from the region of interest over the pharyngeal/laryngopharyngeal and upper oesophageal areas with the fitted pink curve demonstrating a rising pattern for the pharyngeal region. Panel (C) shows the analysis of the supine dynamic study of the liquid gastric emptying time. Panel (D) shows the frequency of reflux to the level of the pharynx/laryngopharynx with the fitted yellow line indicating the residual level after subtraction of background activity

Patients were fasted for 12 hours and medications were ceased for the 24-hours prior to the test. While upright, patients were positioned in front of a Hawkeye 4 gamma camera (General Electric, Milwaukee, USA) with markers placed on the mandible and over the stomach to ensure the regions of interest were within the field of view of the camera. Patients swallowed 100-150 mL of water with 40-60 MBq of ^99m^Tc diethylenetriamine penta-acetic acid followed by flushing with 50 mL of water to clear the mouth and oesophagus of radioactivity. Dynamic images of the pharynx, oesophagus and stomach were obtained for 5 minutes at 15 secs per frame into a 64x64 matrix. A second 30-minute dynamic image was obtained in the supine position immediately following the upright study utilising 30 sec frames (Figure 1). Following acquisition of the supine study, the patients were given a further 50 mL of water with 60 MBq of ^99m^Tc phytate (colloid) followed by 50 mL of water as a flush. Delayed images were obtained at 2 hours to assess the presence of aspiration of tracer activity into the lungs. Images were analysed by time activity curves over the pharynx, upper and lower half of the oesophagus and a background region over the right side of the chest, away from the stomach and oesophagus (Figure 2). Delayed images were analysed by a line profile over the lungs. Time activity curves were graded as showing no GERD, a falling curve, flat or rising curves. Area under the curve and maximal amplitude relative to background were estimated. A liquid gastric emptying half-time was determined from the 30-minute supine acquisition with a single exponential fit to the data.

### Ethical Considerations

Patient data were extracted from a research database of either proven or suspected GERD which had been approved by the Institutional Ethics Committee of Concord Hospital (LNR/12CRGH/248). Patients gave written informed consent for the study under the Institutional Ethics Committee Guidelines.

### Statistical Analysis

All statistics were calculated using the Statistical Package for the Social Sciences Version 24 (IBM, New York, USA). A proportion of the data was non-parametric in nature with ordinal responses such as the isotope time-activity curves for the pharynx and upper oesophagus (Grade 1-3) and lung aspiration of isotope (1=positive, 0=negative). All other variables were parametric or continuous. Spearman rank-order correlation was used for the non-parametric data and Pearson correlation co-efficient was used for the parametric data analysis. The paired t-test was utilised for comparison of test results in the same patient. Binary logistic regression and receiver operating characteristic (ROC) curves were utilised for determining best predictors of lung aspiration of refluxate.

## Results

### Clinical

Results were obtained from 34 consecutive patients undergoing impedance pH/manometric studies and compared with scintigraphic reflux studies. This comprised 15 males and 19 females (mean age: 56 years, range: 28-80 years). The dominant symptoms were of LPR (mainly cough) in 31 and severe typical GERD in 3 cases. The predominant symptom was cough which occurred in 27, other LPR symptoms were recorded in 4, heartburn and regurgitation in 3. Twenty patients were taking PPI, which were ceased for 48 hours prior to testing. No significant differences in results were recorded for the patients taking PPI and those not on PPIs.

A hiatus hernia was present in 16 patients. There was no significant correlation between hiatus hernia and impedance/pH results, manometric or scintigraphic parameters.

### Impedance and pH

**Figure 3:**
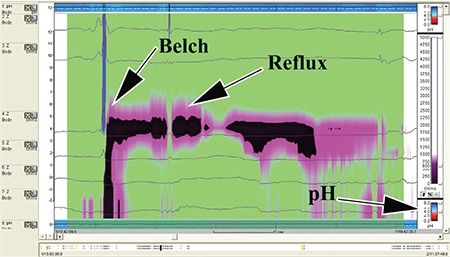
Impedance study demonstrating significant gastro-oesophageal reflux following a belch and the rapid fall in pH and impedance as acid/fluid enters the oesophagus. There is prolongation in clearance of the acid/fluid from the oesophagus (reflux). The pink colour is a marker of the acidity as shown in the colour bar (pH)

Impedance bolus clearance (Figure 3) when upright was a mean of 17.5 [range: 6-42, standard deviation (SD): 7.5] for this population. Results in normal volunteers have been reported as a median of 8 (95% value of 31) (23). Impedance bolus clearance for the 34 patients when supine was a mean of 25.1 (range: 0-214, SD: 34.4), compared to a values of 1-7 in normal volunteers (23). Impedance bolus clearance in total was a mean of 21.1 (range: 8-35, SD: 6.7).

pH results are provided in Tables 1 and 2. The means for acid, non-acid and total proximal reflux were significantly greater than has been reported in normal volunteers by Shay et al. (23). Even the relatively common occurrence of upright reflux in normal volunteers (mean: 1.2%) was significantly higher in this group (mean ~ 10% for acid and non-acid reflux). The frequency of supine reflux at the proximal and distal sites in the oesophagus was markedly greater by an order of magnitude than in normal volunteers (24).

There was no significant difference between proximal acid event frequency and proximal non-acid event frequency in either the upright or supine position by the paired t-test. There was no correlation between any markers of distal pH and either LPR or lung aspiration in the scintigraphic studies.

### Manometric Characteristics of the Group

The LOS pressure was a mean of 2.0 mmHg (range: 0-12, SD: 2.8) compared with a normal sphincter pressures ranging from 18 to 25 mmHg. Thirty patients in this group had a hypotensive LOS. Sphincter pressure was not recorded in 4 patients due to technical difficulty: One patient could not tolerate it and we were unable to traverse the sphincter region in 3 others. Normal oesophageal body motility was present in 4 patients, 9 had a mild non-specific IEM, 4 had moderate IEM and 17 severe IEM according to our modification of the Kahrilas classification (21), where we separated mildly abnormal from normal patients which were included under the normal umbrella in that study.

### Scintigraphy

All 34 patients showed scintigraphic evidence of gastroesophageal regurgitation events and nasopharyngeal contamination in either the upright or supine position or both. A rising or flat time-activity curve was apparent for the pharynx in 30/34 and for the upper oesophagus in 25/34 cases (Table 3). The mean amplitude of activity in the pharynx when compared to background activity of the right upper lung and expressed as a ratio was 4.4 [95% confidence interval (CI): 3.7-5.1].

Pulmonary aspiration of refluxate was apparent in 10 of 34 cases. Of these, 9 patients had atypical histories and 1 had a typical history of heartburn and regurgitation. The most common symptoms associated with aspiration were cough, choking and recurrent throat clearing.

Liquid gastric emptying was abnormal in 12/34 cases (T1/2>16 min). Mean of the abnormal cases was 30 min (95% CI: 15-45 min). There was no significant relationship between liquid gastric emptying and any other scintigraphic results including LPR or lung aspiration of refluxate.

### Statistical Correlations

Impedance and scintigraphy Table 4.

Clearance of refluxate from the oesophagus (impedance bolus clearance) was inversely correlated with the isotope pharyngeal time-activity curves. The longer time to clear the oesophagus of refluxate and return impedance to normal was associated with an increased likelihood of scintigraphic pharyngeal contamination by refluxate and a rising level of refluxate activity in the pharynx in the upright and supine positions (Spearman correlation co-efficient -0.38, p<0.05): Similarly, impaired clearance was strongly associated with increased isotope identification in the upper oesophagus (Spearman correlation co-efficient 0.60, p<0.05). Abnormal gastric emptying appeared to have no association with abnormal oesophageal clearance. There was a strong positive association of all measures of increasing bolus clearance duration and findings of isotope pulmonary aspiration (Spearman correlation co-efficient 0.38-0.60, p<0.05).

Binary logistic regression analysis of pulmonary aspiration found that the best predictor of pulmonary aspiration was the delay in impedance bolus clearance when upright (wald 4.25, p=0.039). Other findings in pH, manometry and scintigraphy did not predict pulmonary aspiration (Table 4).

**Figure 4:**
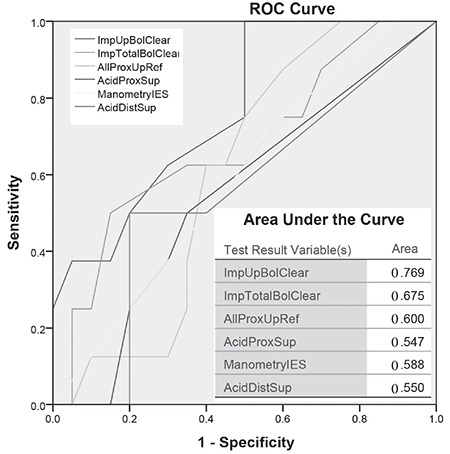
Receiver operating characteristic curve. The curve examines the best predictor of lung aspiration of refluxate amongst the standard testing methods of impedance, manometry and pH studies. The value is based on the comparison of areas under the curve in the interplay between sensitivity and specificity. In this instance, the best predictor of aspiration of refluxate into the lungs are the upright bolus clearance and total bolus clearance in the impedance studies. Note that the least useful value is the acid exposure of the proximal and distal oesophagus in the supine position ROC: Receiver operating characteristic

The best predictor of aspiration of refluxate into the lungs are the upright bolus clearance and total bolus clearance in the impedance studies. This is shown in the receiver operating characteristic curve in Figure 4.

### pH and Scintigraphy

Isotope amplitude in the pharynx was positively correlated with non-acid proximal reflux when supine (Pearson correlation co-efficient 0.35, p=0.04) and all proximal supine reflux (Pearson correlation co-efficient 0.38, p=0.03). A rising curve for the upper oesophagus was associated with significant proximal oesophageal acid exposure. Non-acid proximal reflux when supine was positively correlated with pulmonary aspiration in the scintigraphic studies (Spearman correlation co-efficient 0.36, p=0.04). Proximal acid reflux in the upright or supine position did not correlate with either scintigraphic pharyngeal exposure or lung aspiration of refluxate.

Figure 4 shows that acid exposure in the upper oesophagus was not a good predictor of aspiration of refluxate in the lungs.

### Manometry and Impedance/pH (Reflux/Clearance)

LOS pressure correlated with decreased impedance bolus clearance in the upright position (Pearson correlation co-efficient 0.36, p=0.04) but not in the supine position or with total bolus clearance (Table 5).

A strong correlation was found between decreased manometric LOS pressure and increase in total proximal supine reflux event frequency (Pearson correlation co-efficient 0.58, p=0.001). Total proximal upright reflux event frequency was also correlated with worsening ineffective oesophageal clearance by manometry (Pearson correlation co-efficient 0.40, p=0.02). No significant difference was found between patients with normal oesophageal clearance and those with mild clearance abnormalities and this may be due to inadequate numbers of patients with normal clearance (n=4) leading to a type 1 error.

### Manometry and Scintigraphy

LOS pressure was inversely correlated with isotope amplitude in the laryngopharynx (Pearson correlation co-efficient -0.37, p=0.04) but not with any other scintigraphic variable such as lung aspiration (Table 5).

## Discussion

It is important to state from the outset that the population in this study had severe established gastro-oesophageal reflux disease which was referred to a tertiary centre for consideration of laparoscopic fundoplication, largely after exclusion of other diseases by multiple other disciplines. Nearly all patients had both symptoms and investigative findings consistent with LPR disease. This group was characterized by the failure of response to high-dose PPI therapy. Approximately half the patients had hiatus hernias and the majority, abnormalities of LOS pressures. There was clearly a high susceptibility to high-grade reflux disease in this group of patients as has been reported previously (20). Patients were studied with impedance/pH, manometry and scintigraphic reflux studies. The principal purpose of the studies was assessment for surgery, but these studies have enabled evaluation of the relative contributions of impedance monitoring and standard pH monitoring to predict LPR and lung aspiration of reflux detected by scintigraphic studies, which we have validated in previous work (19,20).

The most outstanding findings in the standard manometric studies was the LOS pressure which was a mean of 2 mmHg compared with a normal range of 18-25 mmHg. Only 4 of 34 patients had normal oesophageal motility underlining the degree of oesophageal clearance abnormalities in this population.

Impedance/pH studies allowed the evaluation of total reflux events (acid + non-acid) in the upper and lower oesophagus in a group of patients in whom PPI and other antacid therapy had been ceased prior to the study. Total proximal reflux measured by the impedance studies was found to be significantly different from both acid (measured by pH probe) and non-acid reflux (measured by impedance probe), reflecting the inadequacy of isolated pH monitoring as a tool for detection of proximal reflux. This is particularly problematic for detection of proximal non-acid reflux which may be significantly more common than acidic reflux (25), given that there is progressive neutralisation of gastric contents as refluxate ascends the oesophagus to its proximal extent or that primary reflux may be non-acidic or even alkaline in patients on maintenance PPI therapy (26,27).

All patients in this study were tested while off PPI therapy. In the study by Mainie et al. (27) on patients with symptoms refractory to PPI therapy (n=144), it was shown that non-acid reflux occurred in 37% and acid reflux in 11% utilising impedance/pH monitoring while still on PPI therapy. In line with these findings was the inverse correlation between LOS pressure and bolus clearance when the patient was upright. It indicates that the severity of abnormal dynamics in the oesophagus cannot be overcome by even favourable gravitational circumstances. A strong positive correlation was also found between total proximal supine reflux event frequency (by impedance probe) and ineffective oesophageal clearance. Not only does the LOS pressure allow free reflux by a mechanical dysfunction but the associated motility disturbance fails to clear the refluxate from the oesophagus. There is a wealth of literature supporting this observation (28,29,30). Impedance bolus transit abnormalities parallel the severity of GERD (31) and in our study showed a significant correlation with a rising upper oesophageal scintigraphic time-activity curve. Under normal circumstances, clearance occurs as a function of gravity and peristalsis with neutralisation of acid by swallowed saliva (30).

The importance of non-acid GERD triggering symptoms has been a vexed issue which has been directly addressed by impedance studies. As the study of Mainie et al. (27) has shown, non-acidic reflux remains a cause of symptoms in patients on high-dose twice daily PPI therapy. Not all patients with non-acidic GERD have symptoms, even patients with established LPR and lung aspiration of refluxate may not have symptoms (20). The lack of symptoms implies a clinically silent but potentially damaging phenomenon and raises the question of appropriate therapy. A number of strategies have been advocated including agents that inhibit transient relaxation of the LOS (32) and experimental endoscopic therapies (26). Ultimately, surgical treatment with laparoscopic fundoplication has efficacy that has been established in numerous studies (19,33,34,35) on the basis of pH monitoring alone and more recently, impedance/pH monitoring in patients with non-acid but symptomatic GERD (36,37).

What additional value does the scintigraphic reflux study contribute to such a group of patients? Impedance and pH studies interrogate both the distal and proximal oesophagus for significant gastro-oesophageal reflux, be it acidic or non-acidic. Distal single-channel 24-hour pH does not show proximal reflux disease as has been shown in this study and dual-channel 24-hour pH is confounded by neutralisation of reflux during ascent of the oesophagus. The combined study assesses the severity and frequency of reflux, forming the basis of principles for treatment. However, the relatively uninterpretable areas that are not reproducibly identified in pharyngeal recording by the combined technique are the laryngopharynx and aspiration of refluxate into the lungs. While there has been some evidence of the utility of impedance studies in the detection of LPR, issues of reproducibility in the pharynx are a significant problem with the current generation of instruments (11). The newer generation of impedance instruments may overcome this problem.

Scintigraphic studies allow direct visualisation of the entry of refluxate into the nasopharyngeal region as a dynamic study in cine format in the upright and supine position (Figure 1). It illustrates whether such activity is rising, static or clearing (Figure 2). We have shown in previous work that a rising pharyngeal curve is highly predictive of lung aspiration of refluxate (19). The delayed study shows if there has been aspiration of tracer into the lungs, which are normally free of tracer activity.

**Figure 5:**
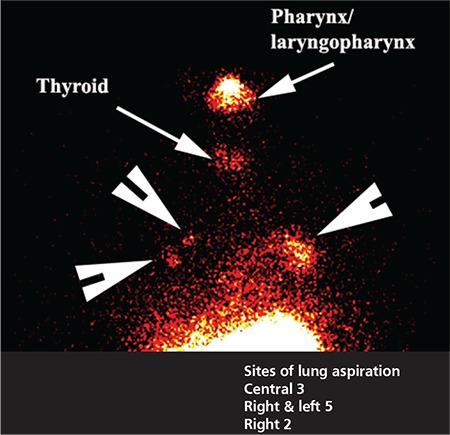
Bilateral lung aspiration. The arrowheads show sites of lung aspiration of refluxate into the main airways in both lungs. Note activity in the pharynx/laryngopharynx and some breakdown of the phytate with free pertechnetate uptake in the thyroid gland (arrows). The Table shows the sites of aspiration which are invariably in the central aspects of both lungs rather than in the lung bases

Scintigraphic studies are a good screening tool but as the sampling time is approximately 33 minutes in total, do not give an actual idea of the overall frequency of GERD, as do the 24-hour recordings of impedance/pH (Figure 3) and manometry. It may well underestimate the frequency and extent of reflux. There is however the clear implication that if such activity is visualised in 30 minutes, under conditions that do not predispose to reflux such as stomach filling as occurs after meals, then it reflects a chronicity of recurrence. Aspiration is screened for at 2 hours (Figure 5). Previous experience with 12-24-hour screening has not added to the pick-up rate significantly and was though not to justify the inconvenience of bringing patients back for a second time. The study has a low radiation dose, being less than a chest X-ray and is relatively non-invasive as the patient swallows approximately 100 mL of radioactive liquid (40-60 MBq). The study is non-invasive and relatively inexpensive to obtain.

All 34 patients showed scintigraphic evidence of pharyngeal contamination with approximately one third aspirating refluxate into the lungs (Figure 5). This is a consistent pattern that we have observed in a previous study of patients undergoing laparoscopic fundoplication for symptomatic GERD and LPR which was resistant to high-dose PPI therapy (19). Time activity curves for the lower pharynx/laryngopharynx and the area under the curve are obtained from a region of interest positioned below retained activity in the oropharynx from the initial swallow (Figure 2). These derived markers of reflux therefore reflect an integration of the volume of reflux and failure of adequate clearance and is an effective parallel of the impedance marker of bolus clearance. The time-activity curves for the region showed a good correlation with the impedance findings of delayed bolus clearance from the oesophagus. The greater the delay in bolus clearance, the more likely was a rising time-activity curve for the lower pharynx/laryngopharynx. Furthermore, the increasing delay in bolus clearance made the chance of pulmonary aspiration much higher. Logistic regression identified the delay in upright bolus clearance as the only factor that predicted pulmonary aspiration of refluxate (Figure 4). This may be an underestimate of supine aspiration, given that aspiration scanning was performed after a period of upright delay, rather than after lying supine for a longer period, as would occur during sleep.

Isotope amplitude is a measure of the highest single reflux episode compared to background activity. It is not a manifestation of “noise” on the curve as it is consistently shown on the curve obtained from the mid/lower oesophagus region of interest in the same temporal sequence. Isotope amplitude for the degree of refluxate in the lower pharynx/laryngopharynx was inversely correlated with LOS pressure and rose with falling pressures. It was also strongly correlated with non-acidic reflux and all reflux in the supine position. Pulmonary aspiration of refluxate was strongly correlated with proximal non-acidic reflux when the patient was supine, suggesting that sleep or the lack of sensory stimulus of less acidic material may disable protective reflex mechanisms (38). Such findings will allow the derivation of a risk-profile that allows prediction of the likelihood of LPR and lung aspiration of refluxate. In contrast, proximal acid reflux did not correlate with either lower pharyngeal/laryngopharyngeal amplitude or aspiration of tracer, again underlining the inherent inability of pH studies in identifying a risk profile for LPR and lung aspiration of refluxate.

## Conclusion

A surprising level of ineffective oesophageal clearance has been identified in this series suggesting that oesophageal body dysfunction is a factor in proximal progression of refluxate. The advent of impedance studies has changed the paradigm for screening patients for GERD. It has brought the issue of non-acidic reflux into focus and increased the understanding of how symptoms can persist while patients are on maintenance high-dose PPI therapy. Many of the findings of oesophageal clearance are well correlated with the scintigraphic reflux studies and allow the formulation of a risk profile for the occurrence of LPR and lung aspiration of refluxate. Scintigraphic reflux studies are a good screening tool for reflux as they also demonstrate extra-oesophageal manifestations in the head, neck and lungs which is spectacularly shown by single photon emission computed tomography fused with low-dose X-ray computed tomography. We have found that LPR and lung aspiration of refluxate can only be attenuated or ceased by surgical fundoplication. Our experience in over 50 cases on maximal medical therapy is that the symptoms may disappear, but LPR and lung aspiration do not.

## Figures and Tables

**Table 1:**
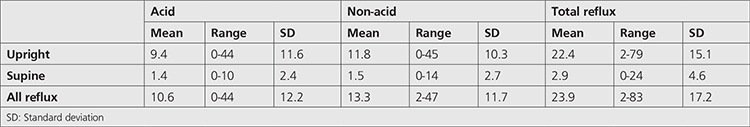
Proximal reflux by pH monitoring

**Table 2:**
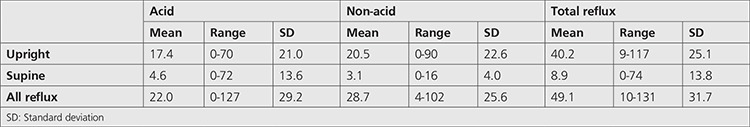
Distal reflux by pH monitoring

**Table 3:**
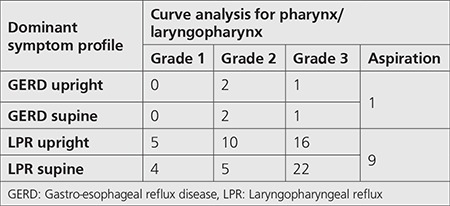
Scintigraphic curve analysis

**Table 4:**
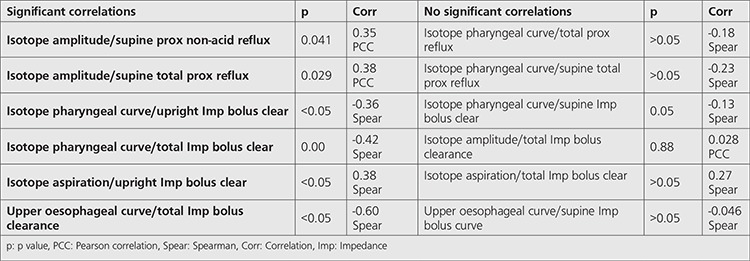
Correlations: Impedance-pH and scintigraphy

**Table 5:**
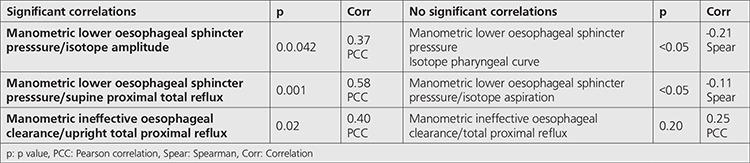
Correlations: Manometry and scintigraphy/Impedance-pH
